# Chronic Hyperglycemia Modulates Rat Osteoporotic Cortical Bone Microarchitecture into Less Fragile Structures

**DOI:** 10.1155/2017/4603247

**Published:** 2017-09-07

**Authors:** Cristina de Mello-Sampayo, Alaíde Alves Agripino, Duarte Stilwell, Bruno Vidal, Ana Luisa Fernando, Beatriz Silva-Lima, Maria Fátima Vaz, Helena Canhão, M. Cristina Marques

**Affiliations:** ^1^Department of Pharmacological Sciences, Faculty of Pharmacy, Universidade de Lisboa, Av. Prof. Gama Pinto, 1649-003 Lisboa, Portugal; ^2^Pharmacological and Regulatory Sciences, iMed, Universidade de Lisboa, Av. Prof. Gama Pinto, 1649-003 Lisboa, Portugal; ^3^MEtRiCS, Unidade de Biotecnologia Ambiental (UBiA), Departamento de Ciências e Tecnologia da Biomassa, Faculdade de Ciências Tecnologia, Universidade Nova de Lisboa, Almada, Portugal; ^4^Clinica Veterinária de Colares, Sintra, Portugal; ^5^Rheumatology Research Unit, Instituto de Medicina Molecular, Faculdade de Medicina da Universidade de Lisboa, Lisboa, Portugal; ^6^IDMEC, Instituto Superior Técnico, Universidade de Lisboa, 1049-001 Lisboa, Portugal; ^7^Nova Medical School and School of Public Health, Universidade Nova de Lisboa, Lisboa, Portugal

## Abstract

There is controversy concerning the diabetes impact on bone quality, notorious in type 2 diabetic postmenopausal women. One pointed cause might be uncontrolled glycemia. In this study, the effect of chronic hyperglycemia in bone turnover, morphology, and biomechanics was evaluated in female Wistar rats in the presence/absence of estrogens (ovariectomy). Animals (*n* = 28) were divided into sham, ovariectomized (OVX), hyperglycemic (streptozotocin 40 mg/kg, single-dose i.p.-STZ), and hyperglycemic-ovariectomized (STZ + OVX) animals. Blood biomarkers were estimated 60 days postovariectomy. Body weight, vertebral microarchitecture (L4-histomorphometry), femur biomechanical properties (bending tests), tibia ultrastructure (scanning electron microscopy), and femur and urinary calcium (atomic absorption) were also evaluated. The increased PINP/CTX ratio of hyperglycemic animals and the similar ratio between STZ + OVX and healthy animals contrasting with the lower ratio of OVX (in line with its histomorphometric data) suggest a tendency for improved bone formation in hyperglycemic-ovariectomized animals. The increased tibia medullar canal, which contrasts with the unaffected cortical thickness of both hyperglycemic groups while that of OVX decreased, was associated to the increased stiffness and strength of STZ + OVX bones compared to those of OVX, in line with the observed ultrastructure. Concluding, chronic hyperglycemia in ovariectomized female rats causes bone morphological changes that translate positively in the ultrastructure and mechanical properties of cortical bones.

## 1. Introduction

The bone structure needs to be stiff, flexible, and light in order to resist biomechanical loading and torsion while allowing movement. These properties are determined by a complex set of interdependent factors, including bone mass, geometrical structure, and tissue composition, which define bone quality and maintain its structural integrity and strength. This overall bone structure is influenced by both the bone ultrastructural properties and bone turnover. The bone ultrastructural properties are directly dependent on the way that bone cells, collagen, and calcium crystals do interact. Bone turnover is an important process for continuous renewal of the cortical and trabecular bone, repair of bone's microdamage and maintenance of mineral homeostasis. Bone cells (osteoclasts and osteoblasts) play an important role in this recycling process by performing bone resortion and formation continuously, whose equilibrium depends on local and endocrinal signals [1].

Osteoporosis is a skeletal disorder characterized by compromised bone strength, linked to ultrastructural modifications that predispose to an increase risk of bone fractures and mortality. There are many known risk factors for osteoporosis and fractures connected to unbalanced bone turnover, but estrogen and androgen deficiencies are the most important causes for primary osteoporosis in elderly individuals of either genders being postmenopausal women particularly affected [2]. Osteoporosis-estimated prevalence based on bone mineral density (BMD) criteria is 11% in women and 2% in men and with increasing prevalence in ageing societies. Moreover, it is estimated that the annual cost of fragility fractures in Europe is €30 billion [3].

The global prevalence of diabetes in 2014 was 8.5% among adults aged over eighteen, and in 2012, a study anticipated that 1.5 million deaths were directly caused by diabetes [4]. Type 2 diabetes (T2DM) is also a chronic disease with high incidence among elderly people and is estimated that like osteoporosis, it will dramatically increase all over the world in the next 15 years [5, 6]. Hypoglycemia and hyperglycemia are daily events even in well-controlled diabetic patients. It is well known that over time, hyperglycemia has a negative effect on several systems, damaging blood vessels and nerves and increasing the risk of heart disease and stroke, retinopathy, neuropathy, infection, and kidney failure. Uncontrolled blood glucose levels may also affect bone quality, but there is still some controversy about the way hyperglycemia affects the bone tissue especially in the case of T2DM [7, 8].

Several reports suggest that T2DM affects both the trabecular and cortical bones increasing the risk for hip, spine, and radius fractures. Although osteopenia has been recognized in T2DM patients pointing to an early screening and prevention of osteoporosis [9, 10], there are many studies that indicate an increase in bone mineral density (BMD) in diabetic postmenopausal women, especially in those with a great body mass index [11, 12]. A recent study showed that there is an association between the increased cortical porosity in T2DM postmenopausal women and fragility fractures [13]. Several factors have been pointed as a major cause of these events; among those are hyperglycemia per se, insulin resistance, high body mass, and ageing response. The effect of chronic hyperglycemia, common in diabetic patients, on bone quality is not yet completely understood. As animal models are valuable tools for studying the bone, the present study aimed to simplify the complexity of the T2DM disease by investigating the effect of one single factor—chronic hyperglycemia—on bone remodeling and ultrastructure and biomechanical properties (strength and stiffness) both in healthy female rats or with reduced levels of 17 beta-estradiol (E2) induced by ovariectomy.

## 2. Materials and Methods

### 2.1. Animals

Female Wistar rats, 2 months of age, from Harlan (Barcelona, Spain) were maintained at an average temperature of 21°C with a 12-hour light/dark cycle and fed with standard laboratory chow (4RF21 LPG, Mucedola Srl, Milan, Italy) and deionized water *ad libitum*. Animal experiments were carried out in accordance with the relevant community and national rules on animals' protection for experimental and other scientific purposes (the EEC Directive (86/609/EEC), the Portuguese laws (DL number 129/92, Portaria number 1005/92) and all following legislations).

### 2.2. Study Design

After 10 days of acclimation, animals were randomly divided into 4 equal groups (*n* = 28): sham-operated (sham group), ovariectomized (OVX group), hyperglycemic (STZ group), and hyperglycemic-ovariectomized (STZ + OVX group) animals. A single dose of 40 mg/kg, i.p. streptozotocin (Sigma-Aldrich, St. Louis, USA) was administered at 2.5 months of age (D −15) to the SZT groups, and glycemia was weekly monitored until 3 months of age (drop of tail blood, using System LifeScan glucometer). At 3 months of age (D 0), both the OVX and STZ + OVX animal groups were ovariectomized by laparotomy under isoflurane anesthesia and the other two groups were sham operated. Preanesthesia (medetomidine hydrochloride 50 *μ*g/kg, i.p. Dechra PLC, UK) and single dose amoxicillin (100 mg/kg, sc, Amoxisol retard, Bayer) and ketoprofen (1 mg/kg, sc, Romefen, Merial Portuguesa) pre- and postlaporatomy were also administered while atipamezole (250 *μ*g/kg, sc, Dechra PLC, UK) was used as sedative reversal. During the recovery period, animals were kept in individual cages. Every 3 weeks, the blood levels of glucose were measured as described above, so that hyperglycemia was monitored in STZ treated rats. Two months (D 60) postovariectomy, all animals were fasted overnight in metabolic cages and urine (12 h) and blood samples were collected just before sacrifice under pentobarbital anesthesia. The femur bones, tibia bones, and lumbar vertebrae were dissected, weighted free of soft tissues, and stored in 70% ethanol (lumbar vertebrae) or at −20°C (femoral and tibia bones).

### 2.3. Analysis of Hormones and Biomarkers

Hormone and biomarker determinations were performed on morning fasting serum samples obtained just before the sacrifice and included the following:

- 17 beta-estradiol (E2) levels determined by monoclonal, competitive, chemiluminescent immunoassay (eE2) according to the guidelines of the manufacturer (using the ADVIA Centaur CP system, Siemens Health Care, Germany) with sensitivity of 11.8 pg/ml
- Metabolic biomarkers, such as glucose, triglycerides (TG), HDL, LDL, and cholesterol, determined by enzymatic assays using an automated analyzer (Olympus AU2700, Hamburg, Germany) and according to the guidelines of the manufacturers (hexoquinase method, GPO/POD system, CHO/PAP system, and cholesterol oxidase, resp.)
- Serum calcium determined on the abovementioned automated analyzer, by the colorimetric Arsenazo III method with sensitivity of 0.13 mg/dl and LoQ of 4 mg/dl

### 2.4. Bone Turnover Marker Quantification

The serum bone turnover biochemical markers, C-terminal telopeptide of type 1 collagen (CTX) as a measure of bone resorption and N-terminal propeptides of procollagen type I (PINP) as a measure of bone formation, were assessed at the end of the study, by enzyme-linked immunosorbent assay (ELISA). Both protein concentrations were determined according to the guidelines of the manufacturer (Immunodiagnostic Systems, Boldon, Tyne and Wear, UK) with sensitivities of 2.0 and 0.7 ng/ml, respectively.

### 2.5. Calcium Content Analysis

The dried (105°C for 12 h) femur bones and urines were digested with nitric acid 65% (PA, Merck, Germany) at 120°C for 4 h and then filtered and diluted to 100 ml of deionized water according to the protocol adapted from AOAC 985.1 [14]. Calcium was measured by atomic absorption spectrophotometry (AAS) (SOLAAR AA spectrometer M series, Thermo Electron Corporation) with acetylene/nitrogen protoxide flame and detection limit of 0.01 mg/l.

### 2.6. Bone Mechanical Testing

**Figure 1:**
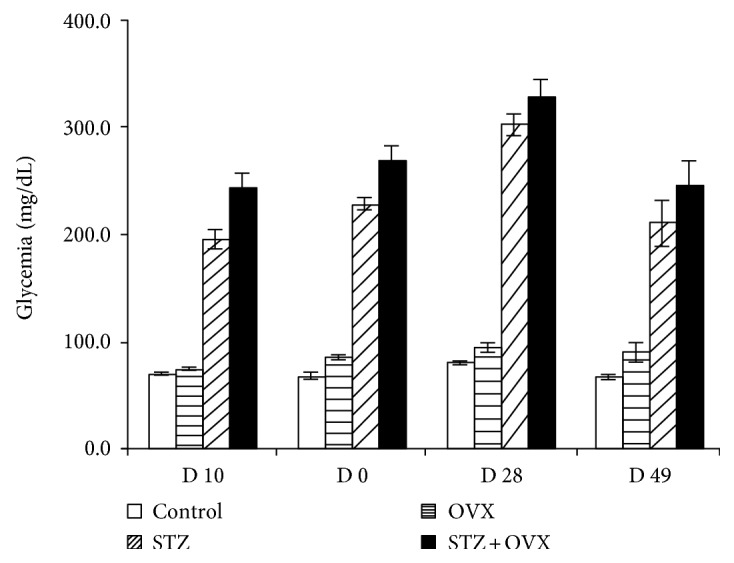
Whole blood glucose level (mg/dl) of studied animal groups during the study: healthy control (sham), ovariectomized (OVX), hyperglycemic (STZ), and hyperglycemic-ovariectomized (STZ + OVX).

The femoral bones, collected at the time of sacrifice, were submitted to three-point bending tests using a universal electromechanical machine (Instron5566™, Instron Corporation, Canton, USA) with a load cell of 500 kN. For the three-point bending tests, the span between the outer loading points was 15 mm with the load being applied to the center of the femoral shaft at a crosshead speed of 0.01 mm/s until the fracture occurred. Stress-strain curves were obtained as previously described [15]. An example of a stress-strain curve obtained is shown in Figure 1. The parameters analyzed from the stress-strain curves were the ones described previously [3, 15], namely, Young's modulus (E), ultimate stress (*σ*u), yield stress (*σ*y), and assuming that femurs were similar to cylinders.

### 2.7. Histomorphometry Analysis of the Trabecular Bone

The 4th lumbar vertebrae (L4) were collected from each animal at sacrifice and stored in 70% ethanol. Bone samples were prepared as previously described [16] with some modifications: samples fixed in ethanol 70% were dehydrated with increasing ethanol concentrations (96% and 100%) and then embedded in methyl metacrylate (MMA) solution. Serial transversal sections through L4 were performed with 5 *μ*m-thickness and stained with Aniline blue in order to distinguish the bone and bone marrow, allowing bone structural analysis. Images were acquired using a Leica DM2500 microscope equipped with a color camera Leica CCD camera (Leica Microsystems, Wetzlar, Germany).

The images of the vertebral trabecular bone sample (L4) obtained at 1.25x magnification were analyzed by a blind expert using a morphometric program (the NIH Image J 1.46R software with plugin Bone J), and parameters such as trabecular separation—Tb.Sp (*μ*m), trabecular thickness—Tb.Th (*μ*m) and ratio of trabecular bone volume/total tissue volume (BV/TV) were evaluated by standard histomorphometric parameters. All variables were expressed and calculated according to the recommendations of the American Society for Bone and Mineral Research [17].

### 2.8. Scanning Electron Microscopy (SEM) of the Tibia Cortical Bone

Longitudinal and transverse cross sections at diaphysis of tibia bone samples were inserted into a mold and mounted in a mixture of *Résine Mecaprex MA2* (04008) and *Durisseur pour Résine Mecaprex MA2* (Presi SA, Tavernolles, 38320 Brie & Angonnes, France) in a 100 : 12 ratio, which gives a transparent resin. After 24 hours, the surfaces of the tibia bone samples were polished using grid papers with designations 200, 320, 600, 800, and 1000, which correspond to different granulometries. Subsequent to the deposition of a uniform conductive layer on the samples, the SEM analysis was carried out in a field emission gun scanning electron microscope (FEG-SEM, model 7001 F, JEOL) using an accelerating voltage of 15 kV. The images were acquired with backscattered electrons. Tibia transverse cross section images obtained at 20x magnification were analyzed using the commercial image analysis software SigmaScan Pro 5 (Sigma Scan software http://systatsoftware.com), and to evaluate the cortical thickness, at least 12 measurements were obtained from each picture. Yet, for the tibia endocortical perimeter measurements, the same images were analyzed using the NIH ImageJ software mentioned in the provision section. A blind expert did also analyze the bone longitudinal cross section images obtained at 150x magnification.

### 2.9. Statistical Analysis

The data are expressed as means and standard error values. Data were analyzed using the nonparametric Kruskal-Wallis test to compare continuous variables. The significance level was set at 0.05. Statistical analyses were performed using the GraphPad Prism.

## 3. Results

### 3.1. Physiological and Biochemical Characteristics of the Ovariectomized Normo-/Hyperglycemic Female Rats

The animals treated with streptozotocin (STZ) had fasting blood glucose levels before laparotomy above 247.4 ± 51 mg/dl. The monitorization of blood glucose levels along the study confirmed the maintenance of the hyperglycemic status of STZ-treated animals (Figure 1) in line with serum glucose levels measured at sacrifice (Table 1). A body weight increase of 18 ± 1% (*p* < 0.05) was observed 2 weeks after STZ administration compared to non-STZ-treated animals (Table 1). At the end of the study, the body weight of ovariectomized (OVX), STZ, and STZ + OVX-treated animals did also increase 24–37.5% compared to that of the sham group. Both groups of STZ animals (STZ and STZ + OVX) showed increase urinary volume compared to non-STZ animals (sham (*p* < 0.01) and OVX (*p* < 0.05)). Serum E2 levels of ovariectomized animals were below the limit of detection (11.8 *μ*g/ml) (Table 1). LDL, HDL, and cholesterol total levels did not show significant changes between the studied groups. However, triglyceride levels increased around 62.5 and 78.1% in STZ animals (STZ and STZ + OVX), compared to ovariectomized normoglycemic and sham animals (*p* < 0.05). Decreased serum calcium levels (*p* < 0.05) along with increased calciuria were observed in OVX (*p* < 0.05) when compared to sham animals. The calcium clearance confirmed the increased calcium loss in OVX animals and in hyperglycemic-ovariectomized animals (*p* < 0.05). Animals' HbA1c was not measured, which can be considered a limitation of the study.

### 3.2. Bone Turnover Markers

The serum marker for bone resorption, CTX, was higher in ovariectomized animals both normo- (65%/69%) and hyperglycemic (64%/68%) as compared to sham and to hyperglycemic nonovariectomized (*p* < 0.05) animals (Table 1). The marker for bone formation, PINP, was higher in hyperglycemic animals (27%) as well as in ovariectomized rats either normo- (46%, *p* < 0.05) or hyperglycemic (71%, *p* < 0.01) when compared to healthy animals (sham). The PINP/CTX ratio increased 22% in hyperglycemic and decreased 14% in ovariectomized while in hyperglycemic-ovariectomized animals, a PINP/CTX ratio similar to that of healthy animals was observed.

### 3.3. Bone Histomorphometry

**Figure 2:**
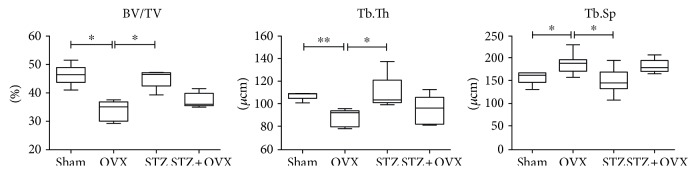
Bone histomorphometry measurements of the fourth lumbar vertebrae (L4) obtained from healthy control (sham), ovariectomized (OVX), hyperglycemic (STZ), and hyperglycemic-ovariectomized (STZ + OVX). Histomorphometry measurements included trabecular occupied area (BV/TV), trabecular thickness (Tb.Th), and intertrabecular distance (Tb.Sp). *n* = 6 per group; ^∗^*p* < 0.05; ^∗∗^*p* < 0.01.

In the ovariectomized rat vertebrae, significant trabeculae thickness decreased (18%/19%) while the intertrabecular distance increased (22%/29%) compared to the vertebrae from healthy (sham) and hyperglycemic (*p* < 0.05) rats (Figure 2). Additionally, the trabeculae area was significantly (*p* < 0.05) lower in bones from ovariectomized rats as compared to healthy and hyperglycemic animals. Moreover, a tendency for improved trabecular bone microarchitecture is observed in the vertebrae from hyperglycemic-ovariectomized rats as compared to the ovariectomized animals (+7.7%, −3.6%, and +9.6% improved Tb.Th, Tb.Sp, and BV/TV, resp.).

### 3.4. Bone Mechanical Properties

**Figure 3:**
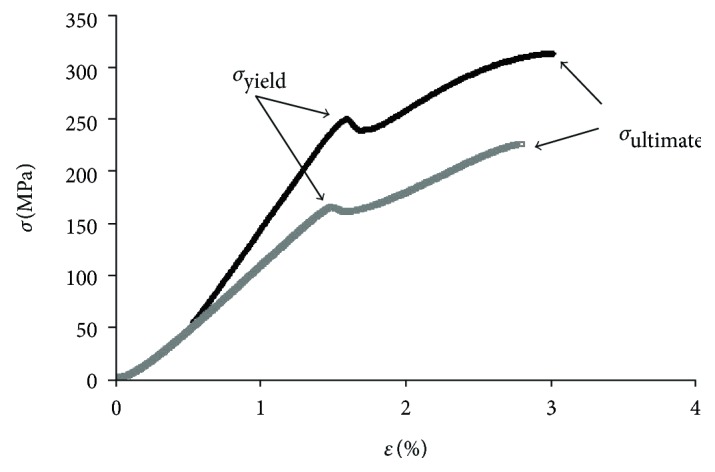
Bone stress-strain curve examples—one for hyperglycemic-ovariectomized (black) and the other for ovariectomized (grey) animals. *σ*_yield_ = yield stress; *σ*_ultimate_ = ultimate stress; ***ε*** = strain.

**Figure 4:**
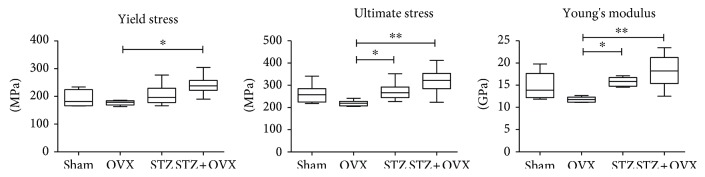
Femur biomechanical properties (yield stress, ultimate stress, and Young's modulus) of the studied female rat groups (healthy control (sham), ovariectomized (OVX), hyperglycemic (STZ), and hyperglycemic-ovariectomized (STZ + OVX)) calculated from mechanical tree-point bending test. *n* = 6 per group; ^∗^*p* < 0.05, ^∗∗^*p* < 0.01.

Stress-strain curves obtained in the three-point bending tests, example given in Figure 3 with pointed yield stress and ultimate stress, were used to calculate the femur biomechanical properties (Figure 4). Each stress-strain curve can be broken down into preyield and postyield portions (Figure 3). Ovariectomized animals showed a tendency for lower values of mechanically measured properties compared to healthy animals. On the other hand, hyperglycemic-ovariectomized animals have femurs with significant higher mechanical properties (*p* < 0.01), namely, elastic modulus (Young's) reflecting 54% higher stiffness; mechanical strength evaluated by the yield stress (36% more force is needed to cause the first microfractures and to start a plastic and definitive deformation of the bone); and ultimate stress (reflecting 46% higher maximum strength of the bone at fracture), compared to femurs belonging to ovariectomized animals. These means that the femur bone from hyperglycemic-ovariectomized animals are less fragile than those from ovariectomized rats as shown in Figure 3. The normalized femur mass and femur diameter of hyperglycemic-ovariectomized animals were not significantly different from those of healthy animals (sham), contrasting with the 26% lower (*p* < 0.001) normalized femur diameter of ovariectomized normoglycemic animals (Table 2).

### 3.5. Bone Mineral Composition

The results obtained by atomic absorption analysis show 22–27% lower calcium content of femur bones from ovariectomized rats either normo- (*p* < 0.05) or hyperglycemic (*p* < 0.01) compared to healthy animals (Table 2). A similar tendency for lower calcium content, although slighter (16%), is observed in hyperglycemic (nonovariectomized) animal femurs.

### 3.6. Bone Cortical Thickness

The analysis of the tibia bone transverse cross section images, obtained at 20x magnification, shows a 7% reduced (*p* < 0.05) cortical thickness and 36% increased (*p* < 0.05) medullary canal in tibia bones from ovariectomized rats as compared to healthy animals while in hyperglycemic animals (ovariectomized or not), the 35–39% increased medullary canal was not accompanied by relevant changes in the bone cortical thickness (0.5–3% reduction) (Table 2).

### 3.7. Bone Structure Evaluation

**Figure 5:**
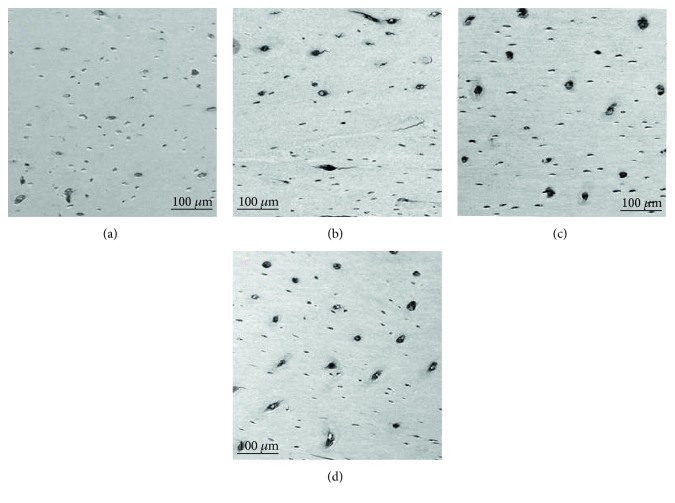
Bone ultrastructure obtained by scanning electron microscopy; images at 150x magnification, of longitudinal tibia cross-sections of healthy controls—sham (a), ovariectomized—OVX (b), hyperglycemic (c), and hyperglycemic-ovariectomized—STZ + OVX (d) animals.

Based on a qualitative evaluation of electronic microscopy of longitudinal tibia cross sections, hyperglycemic animals showed a preserved bone structure with osteons separated by the interstitial bone, lacunae, and osteocytes connected by canaliculus (Figure 5(a)), similar to healthy animals—sham group (Figure 5(b)). However, this group of animals showed an increase in the number and size of bone lacunae when compared to healthy controls.

In contrast, structural bone alterations were identified on both ovariectomized (Figure 5(c)) and hyperglycemic-ovariectomized (Figure 5(d)) groups. Several microcracks, resorptive areas, and increased numbers of lacunae/osteocytes were observed especially in the ovariectomized group.

## 4. Discussion

Although several studies have investigated the association between diabetes and osteoporosis [18–20] and different theories have been proposed [8, 9, 11], the mechanism by which diabetes increases the risk of fractures, independent of BMD, has not been properly identified. The reported increase in fracture risk might be due to factors such as changes in bone geometry and microarchitecture that compromise bone strength and are not reflected in BMD measurements [21, 22] or even to several other factors, among which is chronic hyperglycemia. In this study, we have investigated the effects of chronic hyperglycemia on the bone properties (histomorphology, ultrastructure, biochemical, and mechanic) either in healthy female rats or with reduced levels of E2, induced by ovariectomy.

The rat chronic hyperglycemic status was achieved through partial degeneration of Langerhans islet beta cells induced by a single and smaller dose of streptozotocin [23]. The obtained hyperglycemic rat model showed increase in glycemia, triglycerides, urines' volume, and body weight (compared to sham), at the sacrifice time. By not having induced insulin resistance through high-fat diet [24], this animal model allowed the evaluation of the impact of chronic hyperglycemia (induced by insulin reduction) on bone quality and excluded in part the crosstalk of other complex metabolic factors linked to diabetes condition.

In hyperglycemic animals, the observed tendency for the increased PINP/CTX ratio, mainly supported by the rise in PINP levels, compared to healthy animals, suggests that the permanent hyperglycemia has induced bone turnover through collagen formation predominance. However, the trabecular and cortical thickness of these animals' vertebra and tibia bones, respectively, remained normal. On the other hand, scanning electronic microscopy revealed a preserved bone ultrastructure with an increased number and size of bone lacunae in the tibia cortical bone and a significant increase in the medullary canal when compared to healthy controls. Nevertheless, the mechanical stiffness and strength of the femoral bone from hyperglycemic animals is not affected compared to healthy animals. The effect of diabetes in bone strength and stiffness is not clear yet. Some authors claim that diabetes provides an increased stiffness [25] while others state that a decrease of stiffness is observed [26–29]. In type 1 diabetic rats (body weight loss), the observed diminished bones' stiffness and strength have been attributed to cortical bone growth cessation and to their smaller cross-section size [30] as well as to the bones' resorption increase [27]. On the other hand, but in Zucker Sprague-Dawley model of T2DM [28], the nanoscale morphology of type I collagen was altered with bone mechanical effects at the microscale, suggesting, like in the present study, a matrix more resistant to plastic deformation. As an outcome of bone turnover, collagen fibers align along the direction of the load, which is responsible for an increase in the mechanical strength [31].

Although both type 1 and type 2 diabetes are characterized by hyperglycemia, both have a different impact in bones' structure and mechanical properties. One possible explanation for such different effects on bones could be the glycemia levels (higher in T1DM) and the body weight changes (higher in T2DM) [30]. In addition, it is believed that collagen glycosylation and increase in advanced glycation end products (AGE), which were not quantified in this study, may also play a relevant role behind changes observed in bone microstructure from patients with type 1 or type 2 diabetes and animal models of the disease [9, 28, 32]. Sprague-Dawley rats treated with STZ have significant increased AGE accumulation in the bone [33]. Moreover, spontaneously diabetic rats, both WBN/Kob and BB/Wor/Mol/BB rats, have also increased AGE accumulation in the bone [28]. Since bone fragility increase has been attributed to AGE [34], we can assume that AGE might not be present in relevant amounts in the bones of STZ-treated Wistar rats of this study.

In ovariectomized animals, the serum E2 levels below the limit of detection and the significant increase in body weight after surgery as reported in other studies [35, 36] confirmed the success of the procedure. As expected, ovariectomized animals showed a significant loss of the cortical and trabecular bone as revealed by the trabeculae thickness reduction, the increased intertrabecular distance, and the lower trabeculae area in addition to the reduction of femur diameter and tibia thickness, confirming osteoporosis. These cortical and trabecular thinning results have been reported in other ovariectomized animal studies [36, 37] and in postmenopausal women [38]. Contrariwise to ovariectomized rats, trabecular and cortical bone of hyperglycemic-ovariectomized animals had attenuated losses, which were not significant compared to healthy animals, and presented improved bone structure compared to ovariectomized normoglycemic animals. These results suggest that, at least in rats, the bones of hyperglycemic estrogen-deficient individuals are more protected from the loss of the bone that occurs as a result of the absence of estrogen featuring osteoporosis in postmenopausal women with T2DM.

In humans, the bones of osteoporotic patients have worse mechanical properties, evaluated by the Young's modulus and yield stress, compared to those of healthy controls [40]. The same was observed in rats [36]. In our study, femur biomechanical properties of ovariectomized animals were negatively affected but these changes were only significant when compared to animals under chronic hyperglycemic status ovariectomized or not. In addition, according to the ultrastructure data (SEM), tibia bones from ovariectomized animals have several microcracks, resorptive areas, and an increased number of lacunae but less notorious in those of ovariectomized-hyperglycemic animals. On the other hand, the femur bones of ovariectomized normo- and hyperglycemic animals were less mineralized (lower calcium content) than those of healthy animals and, in a lesser extent, those of hyperglycemic animals too. This can be explained by the increased calcium clearance observed in either ovariectomized normo- or hyperglycemic animals.

All these reported findings suggest that chronic hyperglycemia most possibly is triggering bone microarchitecture modification into less fragile structures, either by the shift of the cortical matrix through the medullary canal enlargement or by the increase in type 1 collagen formation. These bone volumetric changes increase deeply the bone bending strength as recently suggested [22]. Additionally, in *in vitro* studies, when osteoblasts undergo high-glucose concentrations and high-osmotic pressure (mannitol), type 1 collagen secretion increases and reduction of ALP expression [41] indicated both an excess of organic matrix production and a deficit in mineralization capacity as it was observed in the present *in vivo* study. However, the increase in bone lacuna observed in animals under chronic hyperglycemia in this study may, in long term, lead to cortical areas of increased porosity that, when located far from the neutral axis, could confer greater loss of bending strength [21]. This loss of strength could be even more accentuated by loss of bone mineralization. This mechanism could explain, at least in part, the reports [13, 42] that diabetic patients with a higher cortical porosity are most likely to have fragility fractures. Despite the contribution of these findings to further understand the controversies reported in different studies performed in postmenopausal T2DM women, the problem needs to be further explored in future studies to improve the scientific knowledge on the pathogenesis of bone tissue abnormalities in diabetic patients and disclose possible new therapeutic targets and strategies.

Summarizing, chronic hyperglycemia in the female Wistar rats deprived of estrogen causes bone morphological changes that translate positively in the ultrastructure and mechanical properties of the cortical bone, at least on short term.

## Figures and Tables

**Table 1:** Physiological and biochemical markers of the studied animal groups: healthy control (sham), ovariectomized (OVX), hyperglycemic (STZ), and hyperglycemic-ovariectomized (STZ + OVX).

	Sham	OVX	STZ	STZ + OVX
Body weight (g) at D 0	200.31 ± 5.92	206.57 ± 6.53	**239.55** ± **4.42**^a^	**249.16** ± **10.41**^a,b^
Body weight (g) at D 60	218.49 ± 4.97	**274.74** ± **9.98**^aa^	**270.40** ± **10.37**^a^	**300.49** ± **10.41**^aaa^
E2 (pg/ml)	16.50 ± 2.63	<DL	31.89 ± 7.71	<DL
Glycemia (mg/dl)	66.00 ± 15.20	87.33 ± 10.45	**167.33** ± **7.54**^aa,b^	**198.00** ± **21.65**^aaa,bb^
Triglycerides (mg/dl)	31.29 ± 2.29	34.29 ± 3.69	**53.75** ± **5.90**^a^	**57.71** ± **6.07**^a^
Cholesterol (mg/dl)	84.57 ± 3.77	97.00 ± 4.84	90.63 ± 4.77	98.67 ± 4.33
HDL (mg/dl)	39.33 ± 2.46	45.33 ± 2.91	38.2 ± 3.0	37.50 ± 5.86
LDL (mg/dl)	38.17 ± 3.23	49.14 ± 5.57	39.25 ± 4.30	43.71 ± 3.26
Calcemia (mg/dl)	10.47 ± 0.19	**9.76** ± **0.15**^a^	10.26 ± 0.23	9.94 ± 0.29
Urine 12 h (ml)	3.9 ± 0.69	4.3 ± 0.72	**7.2** ± **0.66**^aa,b^	**7.6** ± **0.75**^aa,b^
Calciuria (mg/dl)	31.48 ± 4.47	**65.14** ± **3.29**^a,c,d^	34.68 ± 4.77	37.59 ± 6.13
Ca^2+^ clearance (ml/min)	0.016 ± 0.002	**0.040** ± **0.002**^a^	0.034 ± 0.005	**0.040** ± **0.007**^a^
CTX (ng/ml)	9.38 ± 0.64	**15.45** ± **1.86**^a,c^	9.43 ± 0.91	**15.88** ± **1.43**^a,c^
PINP (ng/ml)	14.66 ± 1.26	**21.33** ± **0.61**^a^	18.58 ± 1.58	**25.01** ± **2.15**^aa^
PINP/CTX	1.57 ± 0.06	1.39 ± 0.11	**1.97** ± **0.0.09**^bb^	1.66 ± 0.13

Average results ± standard error; *n* = 7 per group; ^a^*p* < 0.05, ^aa^*p* < 0.01, ^aaa^*p* < 0.001 compared to sham; ^b^*p* < 0.05, ^bb^*p* < 0.01 compared to OVX; ^c^*p* < 0.01, ^d^*p* < 0.05 compared to STZ + OVX; DL: detection limit = 11.8 pg/ml.

**Table 2:** Bone parameters (femur and tibia) of the studied animal groups: healthy control (sham), ovariectomized (OVX), hyperglycemic (STZ), and hyperglycemic-ovariectomized (STZ + OVX).

	Sham	OVX	STZ	STZ + OVX
Femur bone calcium (%)	14.17 ± 1.05	**11. 07** ± **0.35**^a^	11.96 ± 0.46	**10.31** ± **0.41**^aa^
Femur diameter/BW (*μ*/g)	12.60 ± 0.45	**9.28** ± **0.43**^aaa^	11.03 ± 0.45	10.92 ± 0.51
Femur mass/BW (*μ*g/g)	2.68 ± 0.07	2.58 ± 0.05	2.74 ± 0.06	2.64 ± 0.02
Tibia medullary canal perimeter (mm)	4.63 ± 0.09	**6.31** ± **0.25**^a^	**6.42** ± **0.26**^aa^	**6.27** ± **0.32**^a^
Tibia cortical thickness (mm)	0.636 ± 0.010	**0.592** ± **0.014**^a^	0.633 ± 0.023	0.616 ± 0.016

Average results ± standard error; *n* = 7 per group; ^a^*p* < 0.05, ^aa^*p* < 0.01, and ^aaa^*p* < 0.001 compared to sham.

## References

[1] Fonseca J. E. (2012). Bone biology: from macrostructure to gene expression. *Medicographia*.

[2] Dion N., Ste-Marie L. G. (2012). The fragile beauty of bone architecture. *Medicographia*.

[3] Gonçalves M. J., Rodrigues A. M., Canhão H., Fonseca J. E. (2013). Osteoporosis: from bone biology to individual treatment decision. *Acta Médica Portuguesa*.

[4] World Health Organization (2016). Diabetes mellitus – epidemiology. *Global Report on Diabetes*.

[5] Mathers C. D., Loncar D. (2006). Projections of global mortality and burden of disease from 2002 to 2030. *PLoS Medicine*.

[6] Reginster J. Y., Burlet N. (2006). Osteoporosis: a still increasing prevalence. *Bone*.

[7] Vestergaard P. (2007). Discrepancies in bone mineral density and fracture risk in patients with type 1 and type 2 diabetes - a meta-analysis. *Osteoporosis International*.

[8] Kerckhofs G., Durand M., Vangoitsenhoven R. (2016). Changes in bone macro- and microstructure in diabetic obese mice revealed by high resolution microfocus X-ray computed tomography. *Scientific Reports*.

[9] Wongdee K., Charoenphandhu N. (2011). Osteoporosis in diabetes mellitus: possible cellular and molecular mechanisms. *World Journal of Diabetes*.

[10] Yahuru S., Humphrey S., Landry C., Jain S. K. (2009). Decreased bone mineral density in men with metabolic syndrome alone and with type 2 diabetes. *Medical Science Monitor*.

[11] Rubin R. M., Schwartz A. V., Kanis J. A., Leslie W. D. (2013). Osteoporosis risk in type 2 diabetes patients. *Expert Review of Endocrinology and Metabolism*.

[12] Sta Romana M., Li-Yu J. T. (2007). Investigation of the relationship between type 2 diabetes and osteoporosis using Bayesian inference. *Journal of Clinical Densitometry*.

[13] Patsch J. M., Burghardt A. J., Yap S. P. (2013). Increased cortical porosity in type-2 diabetic postmenopausal women with fragility fractures. *Journal of Bone and Mineral Research*.

[14] Peters J., Combs S., Hoskins B. (2003). *Recommended Methods of Manure Analysis (A3769), University of Wiscosin – Madison*.

[15] Caetano-Lopes J., Nery A. M., Henriques R. (2009). Chronic arthritis directly induces quantitative and qualitative bone disturbances leading to compromised biomechanical properties. *Clinical and Experimental Rheumatology*.

[16] Vidal B., Cascão R., Vale A. C. (2015). Arthritis induces early bone high turnover, structural degradation and mechanical weakness. *PLoS One*.

[17] Parfitt A. M., Drezner M. K., Glorieux F. H. (1987). Bone histomorphometry: standardization of nomenclature, symbols, and units. Report of the ASBMR Histomorphometry Nomenclature Committee. *Journal of Bone and Mineral Research*.

[18] Farr J. N., Drake M. T., Amin S., Melton L. J., McCready L. K., Khosla S. (2014). In vivo assessment of bone quality in postmenopausal women with type 2 diabetes. *Journal of Bone and Mineral Research*.

[19] Giangregorion L. M., Leslie W. D., Lix L. M. (2012). FRAX underestimates fracture risk in patients with diabetes. *Journal of Bone and Mineral Research*.

[20] Schwartz A. V., Vittinghoff E., Bauer D. C. (2011). Association of BMD and FRAX score with risk of fracture in older adults with type 2 diabetes. *The Journal of the American Medical Association*.

[21] Pritchard J. M., Giangregorio L. M., Atkinson S. A. (2013). Changes in trabecular bone microarchitecture in postmenopausal women with and without type 2 diabetes: a two year longitudinal study. *BMC Musculoskeletal Disorders*.

[22] Seeman E. (2015). Growth and age-related abnormalities in cortical structure and fracture risk. *Endocrinology and Metabolism*.

[23] Akbarzadeh A., Norouzian D., Mehrabi M. R. (2007). Induction of diabetes by streptozotocin in rats. *Indian Journal of Clinical Biochemistry*.

[24] Skovso S. (2014). Modeling type 2 diabetes in rats using high fat diet and streptozotocin. *Journal of Diabetes Investigation*.

[25] Funk J. R., Hale J. E., Carmines D., Gooch H. L., Hurwitz S. R. (2000). Biomechanical evaluation of early fracture healing in normal and diabetic rats. *Journal of Orthopaedic Research*.

[26] Einhorn T. A., Boskey A. L., Gundberg C. M., Vigorita V. J., Devlin V. J., Beyer M. M. (1988). The mineral and mechanical properties of bone in chronic experimental diabetes. *Journal of Orthopaedic Research*.

[27] Glorie L., Behets J. G., Baerts L., De Meester I., D'Haese P. C., Verhulst A. (2014). DPP IV inhibitor treatment attenuates bone loss and improves mechanical bone strength in male diabetic rats. *American Journal of Physiology. Endocrinology and Metabolism*.

[28] Hammond M. A., Gallant M. A., Burr D. B., Wallace J. M. (2014). Nanoscale changes in collagen are reflected in physical and mechanical properties of bone at the microscale in diabetic rats. *Bone*.

[29] Verhaeghe J., Suiker A. M. H., Einhorn T. A. (1994). Brittle bones in spontaneously diabetic female rats cannot be predicted by bone mineral measurements: studies in diabetic and ovariectomized rats. *Journal of Bone and Mineral Research*.

[30] Silva M. J., Brodt M. D., Lynch M. A. (2009). Type 1 diabetes in young rats leads to progressive trabecular bone loss, cessation of cortical bone growth and diminished whole bone strength and fatigue life. *Journal of Bone and Mineral Research*.

[31] Caetano-Lopes J., Nery A. M., Canhão H. (2010). Chronic arthritis leads to disturbances in the bone collagen network. *Arthritis Research & Therapy*.

[32] Reinwald S., Peterson R. G., Allen M. R., Burr D. B. (2009). Skeletal changes associated with the onset of type 2 diabetes in the ZDF and ZDSD rodent models. *American Journal of Physiology. Endocrinology and Metabolism*.

[33] Reddy G. K., Stehno-Bittel L., Hamade S., Enwemeka C. S. (2001). The biomechanical integrity of bone in experimental diabetes. *Diabetes Research and Clinical Practice*.

[34] Karim L., Bouxsein M. L. (2016). Effect of type 2 diabetes-related non-enzymatic glycation on bone biomechanical properties. *Bone*.

[35] Kalu D. N. (1991). The ovariectomized rat model of postmenopausal bone loss. *Bone Mineral*.

[36] Zhao Q., Zhang L., Shen X. (2013). Bone selective protective effect of a novel bone-seeking estrogen on trabecular bone in ovariectomized rats. *Calcified Tissue International*.

[37] Muhammad N., Luke D. A., Shuid A. N., Mohamed N., Soelaiman I. N. (2012). Two different isomers of vitamin E prevent bone loss in postmenopausal osteoporosis rat model. *Evidence-based Complementary and Alternative Medicine*.

[38] Seeman E. (2008). Structural basis of growth-related gain and age-related loss of bone strength. *Rheumatology*.

[39] Starup-Linde J., Lykkeboe S., Gregersen S. (2016). Bone structure and predictors of fracture in type 1 and type 2 diabetes. *The Journal of Clinical Endocrinology and Metabolism*.

[40] Ciarelli T. E., Fyhrie D. P., Schaffler M. B., Goldstein S. A. (2000). Variations in three-dimensional cancellous bone architecture of the proximal femur in female hip fractures and in controls. *Journal of Bone and Mineral Research*.

[41] Cunha J. S., Ferreira V. M., Maquigussa E., Naves M. A., Boim M. A. (2014). Effects of high glucose and high insulin concentrations on osteoblast function in vitro. *Cell and Tissue Research*.

[42] Choi Y. J., Chung Y.-S. (2016). Type 2 diabetes mellitus and bone fragility: special focus on bone imaging. *Osteoporosis and Sarcopenia*.

